# Medical Obituary

**Published:** 1840-12

**Authors:** 


### MEDICAL OBITUARY.

Dudley Woodbidge Rhodes, M. D. It is with grief that we record the death of this gentleman,—a talented physician and one of the most estimable citizens of Zanesville, Ohio. Dr. Rhodes was a native of Stonington, Connecticut, and studied his profession in Hartford, under the direction of the late and much respected Dr. Mason F. Coggswell, the projector of the Asylum for the Deaf and Dumb in that city. During the last war with England, Dr. Rhodes entered the medical staff of the Army, and afterwards emigrated to Zanesville, where he continued in the diligent practice of his profession, to the time of his death, on the 18th of October last. Our acquaintance with him commenced in the autmn of 1815 when, a young man, he was but beginning his labours, in the town, where he afterwards became distinguished and beloved. He was then, what every young physician should be, amiable, benevolent, cheerful, temperate, studious, and devoted, in soul and body, to the interests of those who employed him. The fruits of these *semina* of character, were what they must always be, the esteem and respect of the community, its confidence, its patronage, and its grief when he died.

We saw Dr. Rhodes in the month of August last, when he did not appear to be in good health, but was diligently occupied in the duties of his profession. In this occupation he continued until about the 10th or 11th of October. On the 12th he sent for his faithful friend and pupil Dr. Moorehead, who, as he informs us, found him in his office complaining of an excruciating pain in the forehead, with a firm pulse. Dr. M. proposed blood-letting, but his patient objected, and the next day visited a patient in the country and several in the city. On the 14th he was worse and kept his bed most of the day. On the 15th he was lethargic, and another medical friend, Dr. Safford of Putnam, was called into consultation. As it is not our design to report his case in this notice, we shall only add, that on the morning of the 18th his symptoms were decidedly apoplectic, and that he expired at one o'clock.

A *post mortem* examination of the brain was made and disclosed an abcess in each anterior lobe of that organ, containing about an ounce of pus, with decided softening of the surrounding cerebral substance.

Referring to these manifest ravages of inflammation, Dr. M. remarks—"I cannot but regret the misfortune of not being able to prevail on my patient to permit the active treatment which I proposed on my first and immediately subsequent visits. Most of the symptoms except the cephalalgia, were of such a mild and obscure character that he could with difficulty be prevailed upon to do any thing of an effective character. Physicians, generally, as far as my observation has gone, are unmanageable patients; and no class of men are more likely to deceive themselves. I doubt, however, whether any plan of treatment, adopted after I was called in, would have saved him, as his family inform me, that for some weeks previous to his being taken down, he frequently complained of headache, and that his memory was impaired. Thus, it would appear, there was a morbid action in his brain, for a considerable time before any treatment commenced."

In this opinion we concur, from having observed in visiting several patients with our deceased friend, that his memory of the symptoms and treatment of the preceding day was imperfect. To this enfeeblement of mind, the direct consequence of his cerebral disease, we may ascribe his error in refusing to submit to the only treatment which could have saved him.

On the day of his interment the physicians of Zanesville, and its vicinity, held a meeting of which Dr. Mitchell was chairman and Dr. Hildreth secretary, when the following preamble and resolutions were unanimously adopted:

"Whereas it has pleased the Almighty to remove from amongst us, and from the sphere of his usefulness, our late friend and brother practitioner, Dr. D. W. Rhodes: therefore,

"*Resolved*, That we view with deep regret this dispensation of Divine Providence, inasmuch as it has borne from us one, who has for many years stood so deservedly high in his profession; and who has been most indefatigable in his exertions to alleviate the sufferings of his fellow-men.

"*Resolved*, That in the death of Dr. Rhodes this community has sustained a loss, which will be most severely felt by those who in the hour of affliction, have so long relied upon his skill and judgment.

"*Resolved*, That we deeply sympathize with his bereaved family, in their painful and melancholy loss.

"*Resolved*, That in testimony of our regard for the deceased, we wear crape on the left arm for thirty days.

"*Resolved* That a copy these resolutions be presented by Dr. Moorehead to the family of the deceased, and that they also be published in the papers of this town.

"*Resolved*, That we adjourn to meet at the late residence of Dr. Rhodes, at the hour appointed for his funeral, and that we attend the same in body."

This is a merited tribute, and it is delightful to find it so willingly given by surviving brethren.

John A. Turner, M. D. A few weeks before the death of Dr. Rhodes, the profession and people of Zanesville had experienced the loss of another intelligent and honorable physician, Dr. Turner. He was a native of the valley of Virginia, where, in the memorable epidemic, autumnal fever of 1823, he experienced an attack, which left him with an enlarged spleen. From this resulted repeated attacks of intestinal hæmorrhage, followed at last by ascites, of which he died. We have a full history of his case taken from his own mouth, with an account of the autopsic appearances, communicated to us by Mr. Hazlett, a student of medicine, which presents so many points of interest, that we shall lay it before our readers in a future number. Dr. Turner was greatly beloved by his acquaintances, and died amidst the regrets of society. D.

